# Digital Empowerment for Older Women: Addressing Inequality Through Competence Training

**DOI:** 10.3390/healthcare14040489

**Published:** 2026-02-14

**Authors:** Sinem Burcu Uğur, Nehir Yasan-Ak, Aylin Çiçekli

**Affiliations:** 1 Department of Social Work, Manavgat Faculty of Social Sciences and Humanities, Akdeniz University, Antalya 07058, Türkiye; sinemburcu@akdeniz.edu.tr; 2 Department of Management Information Systems, Manavgat Faculty of Social Sciences and Humanities, Akdeniz University, Antalya 07058, Türkiye; 3 Department of Sociology, Faculty of Letters, Akdeniz University, Antalya 07058, Türkiye; aylincicekli@akdeniz.edu.tr

**Keywords:** digital competence, digital capital, empowerment, digital inclusion, social participation, older women, active aging

## Abstract

**Background/Objectives:** Digitalization creates new opportunities for social participation and access to services; however, individuals who lack access to digital resources or the ability to use them effectively are often unable to benefit from these developments. This uneven distribution reflects differences in digital capital that enhance technical competence and support psychosocial dimensions, contributing to empowerment. Such inequalities are particularly pronounced where age- and gender-based disadvantages intersect, generating distinctive forms of exclusion and vulnerability. Within the framework of digital capital theory, this study aims to explore how older women learners’ digital capital is shaped through a structured Digital Competence Training Program and how its growth influences their empowerment experiences. **Methods:** This qualitative case study utilized semi-structured interviews with 13 older women learners, two policymakers, and an educator. **Results:** Learners achieved gains beyond technical skills, including greater autonomy, self-confidence, and social connectedness. Despite technical and structural limitations, participants described the program as transformative, strengthening competence and belonging. **Conclusions:** The training program is an important pathway for strengthening older women’s digital capital and fostering empowerment beyond technical skill acquisition. However, its transformative potential depends on broader structural and institutional conditions, underscoring the need for inclusive, community-based digital education policies to sustain digital participation in later life.

## 1. Introduction

Active aging policies emphasize not only health and security, but also the ability of older people to live autonomously, productively, and as active participants in society. Within this framework, supporting older adults’ social participation is as critical as addressing their physical needs [1,2]. Digital technologies enhance the visibility and participation of older adults in social and public life, offering significant opportunity for engagement [3,4]. However, access to digital tools and the ability to use them effectively remain significant sources of inequality for many older people [5]. The interplay of age, gender, and socio-economic disadvantages shapes older adults’ lives [6,7]. Older women are among the most disadvantaged, facing restricted access to resources, weaker positions in education and social security, and persistent gender norms [8]. These inequalities extend to the digital sphere, where older women encounter disproportionate barriers to access and use, placing them at the center of digital exclusion [9,10]. Although recent evidence from Germany indicates that gendered digital divides in later life are gradually narrowing [11], in Türkiye they remain pronounced, underscoring the need for context-specific analyses.

Recent national statistics further illustrate the depth of the gendered digital divide in Türkiye. According to the Turkish Statistical Institute’s (TurkStat) 2025 Household Information Technologies Usage Survey [12], while overall internet use among individuals aged 16–74 reaches 90.9%, usage drops sharply to 29.6% among those aged 65–74. Within this age group, device ownership remains highly gendered, with 39.3% of women owning digital devices compared to 55.3% of men. Similarly, although 76.1% of the general population uses e-government services, this figure falls to 29.6% among adults aged 65–74, with women using such services 13.3 percentage points less than men. These figures underscore the compounded effects of age and gender in shaping digital exclusion among older women in Türkiye. These patterns suggest not only a persistent gendered digital gap, but also a lack of context-sensitive understanding of older women’s digital lives; while aggregate statistics reveal structural inequalities, they offer limited insight into how these inequalities are experienced on a day-to-day basis. However, research exploring the lived realities behind these figures remains limited, and while international research has recognized digital technologies’ role in fostering social participation and active aging [3,13,14], studies in Türkiye remain limited. Existing work, mostly quantitative, tends to define digital competence narrowly as technical skill, overlooking its social, psychological, and structural dimensions [15,16]. In particular, there is a striking absence of qualitative research focusing on older women’s digital experiences in Türkiye, despite their intersectional disadvantages related to age, gender, and socio-economic positioning. In the Turkish context, the limited scholarly attention given to older women is closely linked to the social construction of age and gender. Research on aging and gender in Türkiye shows that older women are often positioned within normative expectations of dependency and domesticity, with caregiving roles becoming more pronounced in later life [17,18]. As these age- and gender-based norms orient older women more strongly toward the private sphere, their visibility in public, social, and increasingly digital domains tends to decrease, heightening the risk of social isolation and rendering their everyday experiences less likely to be examined through qualitative research.

Addressing this gap in the gender and aging literature, the present study seeks to understand older women’s digital engagement not only in terms of technical competence, but also as a process shaped by social positioning, everyday practices, and experiences of empowerment. In this context, the Digital Competence Training Programme (DCTP) was previously developed in response to the findings of a previous Scientific and Technological Research Council of Turkey (TÜBİTAK)-funded project that revealed profound inequalities in older women’s digital access and use in Türkiye [19]. The DCTP was implemented as a structured, community-based initiative targeting older women registered in municipally affiliated older adult centers in Antalya. The present study examines how the DCTP contributed to the accumulation of digital capital among older women and how this capital was subsequently reflected in their everyday lives and social participation. More specifically, it explores how perceived deficits in digital capital motivated participation in the training programme and how these resources were subsequently mobilised in daily practices.

To capture this multidimensional process, the analysis is guided by Ragnedda and Ruiu’s [20] concept of digital capital, which in this study is examined through two interrelated components—digital ownership and digital competence—following Ragnedda and Ruiu’s framework. Digital ownership refers to access-related conditions, including possession of digital devices, internet connection, time spent online, and access to support or training. Digital competence, in turn, encompasses a set of interrelated abilities, including information and digital literacy, communication and collaboration, digital content production, security, and problem-solving. Analytically, the study distinguishes between the technical, social, and psychological implications of these competences as they are experienced by older women. This analytical distinction does not imply that these dimensions are separate. Rather, it helps illuminate how digital competences function simultaneously as skills, social resources, and sources of autonomy in everyday life. This approach highlights how digital competences are embedded within social structures and how they intersect with broader inequalities [21,22,23], making it well-suited for analysing the layered effects of digitalization on older women’s lives. This understanding closely aligns with the aims of the DCTP, which sought to develop not only technical abilities but also the confidence, safety awareness, and practical capacities required for meaningful digital engagement. Accordingly, digital capital provides an appropriate analytical framework for examining how participation in the training programme contributes to the development of these competences and how they are subsequently mobilised in older women’s everyday lives. On this basis, the study addresses the following research questions:

RQ1: What motivations and perceived deficiencies shaped older women’s participation in the DCTP?
RQ2: How did the programme influence different dimensions of older women’s digital capital?
RQ3: In what ways did changes in digital capital affect older women’s everyday practices, social relationships, and forms of social participation?

This study contributes to the literature in three key ways: (1) it provides a rare qualitative insight into older women’s digital lives in Türkiye; (2) it explores digital skills as social and empowering tools; and (3) it applies digital capital theory to unpack how inequalities are reproduced or transformed in later life. Building particularly on the second and third contributions, this study advances digital capital theory by showing that digital competences—understood as more than basic digital literacy—function not only as technical skills but also as sources of confidence, visibility, and social participation. In later life, these competences operate as symbolic and relational forms of capital.

## 2. Materials and Methods

This qualitative case study, conducted in Antalya, Türkiye, explores how digital competence training shapes older women’s digital capital and, in turn, their participation in social life.

### 2.1. An Overview of the DCTP

The DCTP consisted of five modules focusing on: (1) the use of information and communication technologies (smartphones, tablets, and computers); (2) content creation, including email use and social media; (3) digital services, such as accessing and completing online applications; (4) access to reliable information, including information verification and emergency communication; and (5) digital security, with an emphasis on safe internet use and identity protection. Training activities were conducted using participants’ own smartphones as well as tablets provided to them for the duration of the programme, which remained with participants and offered continuous internet access. The programme was delivered over an eight-week period and combined face-to-face sessions with online instruction. Pedagogically, it adopted a practice-oriented and learner-centred approach with application-based training, continuous instructor guidance, and opportunities for peer support that encouraged active and collaborative learning. Through this structure, the programme sought to support older women’s independent, confident, and safe engagement with digital technologies in everyday life.

### 2.2. Participants

A multi-actor perspective was adopted to capture both the individual and structural dynamics of digital empowerment. The fieldwork involved 13 older women learners aged 60 and above, alongside two policymakers and one educator; while the learners provided first-hand accounts of their digital experiences, the educator and the policymakers contributed complementary perspectives based on their roles in the design and delivery of the programme, as well as their observations of its outcomes.

The learners’ ages ranged from 60 to 78 years (M=68.69; SD=5.53). Most were married and had children, situating them predominantly in maternal and grandmaternal roles. Educational backgrounds ranged from primary school to master’s level, with secondary education being the most common. Ten learners were retired, having previously worked in the public or private sector, while three primarily identified as housewives. Although income data were not formally collected, participants resided in urban neighbourhoods in Antalya and had varying employment and education histories. These characteristics suggest a predominantly lower- to middle-income profile. These socio-demographic features are not presented solely as background variables, but rather as factors influencing participants’ digital needs, motivations, and access patterns throughout the study.

### 2.3. Data Collection and Procedures

The DCTP was not designed as an open-access intervention for all adults aged 60 and over, but was primarily intended for women who were members of municipally affiliated older adult centers. In Türkiye, such centers operate alongside residential care and nursing facilities but function as community-based spaces that promote social participation, lifelong learning, and well-being. In Antalya, where the study was conducted, five municipally affiliated older adult centers operate within this framework. Although the programme was open to women attending any of these centers, participation was voluntary and not center-wide. The DCPT reached approximately 60 learners across all training cycles. As some women participated in more than one cycle, the total number of unique learners was approximately 50. Using purposive sampling, of the approximately 50 women who completed the DCTP, only those meeting the interview eligibility criteria were invited to participate in the qualitative study. Eligibility required: (1) completion of the DCTP, (2) current residence in Antalya, (3) the cognitive and physical ability to meaningfully participate in an interview, and (4) provision of informed consent. Although no formal cognitive screening tools were used, the course educator informally assessed participants’ readiness based on prior interaction during the programme. Individuals undergoing intensive medical treatment or experiencing advanced cognitive impairment were not included. Prior digital exposure was not used as an inclusion or exclusion criterion, as the study aimed to capture a diverse range of digital experiences. In total, 13 women who met all criteria were interviewed.

To enrich the analysis, the study also included interviews with the programme’s educator and two policymakers, capturing both design perspectives and implementation experiences; while learners reflected on their motivations, learning processes, and perceived outcomes, the educator and the policymakers provided insights into pedagogical intentions, implementation practices, and institutional rationales underlying the programme. These perspectives were analysed comparatively to identify alignments, gaps, and tensions between programme design, delivery, and lived experience, enabling a more nuanced interpretation of how digital capital was intended, experienced, and supported within the programme context.

The data collection process was conducted through semi-structured, in-depth interviews carried out jointly by the second and third authors between August and October 2024. The interview questions were developed in line with the aims of the study and were organised around a common set of analytical themes. While the core questions remained conceptually similar across participant groups, they were adapted in wording and perspective to reflect each participant’s role in the programme. For older women learners, questions focused on personal motivations for enrolling in the programme, expectations regarding digital learning, and perceived outcomes of participation and willingness to continue or recommend the programme. Sample questions included, for example, “What motivated you to participate in the programme?” and “What changes, if any, did you experience in your daily life following the training?” For the educator and the policymakers, the same themes were explored based on their direct involvement in the programme, their observations during the training process, and their familiarity with findings from the related the TÜBİTAK project.

In total, 16 interviews were conducted, each lasting between 30 and 60 min, with an average duration of approximately 40 min. All interviews were audio-recorded with participants’ informed consent and transcribed verbatim for analysis. As the study is based on qualitative interviews, the findings reflect participants’ self-reported experiences and interpretations. The research design did not include observational measures or standardised pre–post assessments of digital competence; instead, changes in digital engagement and perceived outcomes were examined through participants’ narratives. This study adhered to institutional research ethics guidelines and received approval from the Scientific Research and Publication Ethics Committee of Akdeniz University on 14 December 2023 (Approval No. 803436). Participation was voluntary, and all participants were informed about the study’s purpose, procedures, and their right to withdraw at any time without consequence. No identifying information was collected, and confidentiality was ensured through secure data storage accessible only to the research team.

### 2.4. Data Analysis

Data were analyzed using thematic analysis [24]. The process was fully inductive; no prior coding scheme or theoretical framework was applied, and no literature review was conducted prior to analysis. The semi-structured interviews were audio-recorded using an Olympus WS-852 digital voice recorder (Olympus Corporation/OM Digital Solutions Corp., Tokyo, Japan) and subsequently transcribed into text using the Transkriptor version 2.1.14 software (Textintel FZE, Dubai, United Arab Emirates). All transcripts were manually reviewed and corrected for accuracy by the researchers. The initial codes were derived directly from the repeated patterns and expressions in the interview data. Coding and data management were conducted manually using Microsoft Word and Excel (Microsoft Corporation, Redmond, WA, USA). Although one researcher led the coding process, all three researchers independently reviewed the transcripts, created initial codes, and compared findings to check for consistency. Discrepancies in interpretation were discussed collectively until consensus was reached; while formal inter-coder reliability metrics were not used, all transcripts were independently reviewed and cross-checked by three researchers to enhance credibility. Through this iterative process, overlapping codes were consolidated into broader categories. This led to the identification of four key themes: digital capital, motivation, perceived outcomes, and structural barriers.

## 3. Results

Qualitative data were analyzed through Ragnedda and Ruiu’s [20] digital capital framework, drawing on a multi-actor perspective that included the experiences of older women as well as the insights of educators and policymakers. The findings were thematically organized into four categories—digital capital, motivations, gains, and barriers—based on the framework’s analytical dimensions and research questions. Each theme is presented through this tripartite lens, explicitly acknowledging heterogeneity among participants. Notably, among the older women learners’ experiences were shaped by considerable heterogeneity in age, prior digital exposure, and socio-economic background. These factors influenced learning trajectories and reported outcomes by affecting the pace of learning, confidence levels, and the types of digital practices they considered most useful or meaningful in their daily lives.

### 3.1. Theme 1: Digital Capital

The implementation of the DCTP was primarily informed by findings from the TÜBİTAK-supported project (No. 120k613), which documented low levels of digital capital among older women. These findings provided the main rationale for implementing the DCTP. The policymakers and the educator were already aware of these limitations through the previous project and designed the training accordingly. Learners confirmed this diagnosis by making the same problems visible in their own experiences. Across learners, the educator, and the policymakers, there was a shared understanding that older women entered the programme with limited digital capital, which serves as the analytical starting point for this theme. Drawing on Ragnedda and Ruiu’s [20] definition, the digital capital of trainees was considered through two fundamental dimensions: digital ownership and digital competence. These are presented in this theme as two sub-themes, making visible the “initial level” prior to the program.

#### 3.1.1. Sub-Theme 1a: Digital Ownership

Digital ownership refers to individuals’ access to digital devices and connections. The policymakers and the educator emphasized that older women’s ownership was almost negligible. While smartphone ownership appeared widespread, most devices were outdated with limited features; regular internet access was inconsistent, and access to computers or tablets was rare. Ownership was not only a starting point but also a prerequisite for developing competence, as hardware and internet access were essential for digital learning:

“Older women are often citizens who cannot use digital devices or access the internet. Since they lack ownership at the first stage, they already fall far behind at the second stage—namely, using devices and developing competence.”

(PM1)

Therefore, throughout the program, all learners were provided with a tablet with 24/7 internet access, allocated exclusively for their use, and the training was delivered through these devices (the tablets were supplied by a private-sector partner of the program). The learners’ accounts largely confirmed this assessment. At the time of the interviews, all learners reported that the tablets with 24/7 internet access provided during the programme had been retrieved upon its completion, and that they owned a smartphone as their primary personal digital device. A small number also reported owning additional devices, including desktop (n=2) and notebook computers (n=2), with one participant owning both; however, these devices were described as being used rarely or not at all in daily life. While many reported continuous internet access, usage was limited. Some trainees (L1, L4, L8) integrated the internet into daily life, while others (L3, L5, L7, L9, L12, L13) used it only occasionally, reporting 2–3 h or less per day. Outside the DCTP, experiences with digital learning were almost entirely informal. Only L4 had attended a computer course. Others developed skills through trial and error or with family support (L1, L5, L9, L11). Children and grandchildren were the most common sources of help, though some women lacked any support at all. In summary, while device ownership existed, it remained qualitatively limited, shaped by dependencies and low-intensity use.

#### 3.1.2. Sub-Theme 1b: Digital Competence

Participants’ digital competences were explored through five dimensions identified in the digital capital framework: digital literacy, communication and collaboration, content creation, safety, and problem-solving. According to the policymakers and the educator, older women’s competences were highly limited. Even when they overcame device access barriers, they struggled to use them independently, securely, and effectively. Learners similarly acknowledged their low competences prior to training. The majority of learners reported some familiarity with the digital environment, but their use was mostly confined to basic functions such as news, recipes, or knitting patterns. Communication was the most frequent use, largely through WhatsApp for messaging and sharing visuals. Social media was also used, but mainly for consumption. Only a small group (n=5) engaged in sharing or commenting, usually limited to photos or short videos. Some (L1, L2, L4) described their smartphones as “ornamental objects” outside of these functions. Conceptual gaps were also evident. For instance, some trainees (L3, L7) used practices requiring internet but replied “No” when asked if they had internet. The weakest competences were in safety and problem-solving. With the exception of one learner, all learners avoided services such as online banking, e-government, and digital health platforms. For most learners (n=8), avoidance was shaped by perceived risks and uncertainty, while others articulated more personalised concerns related to confidence, control, and trust in digital environments. As L5 put it:

“I worry about entering the wrong site. My son is an IT trainer, and I trust him, but I do not dare to use it on my own.”

(L5)

Similarly, L9 noted that she avoided online shopping because she did not know how to recognize secure sites. These examples suggest that women’s reluctance stemmed less from a lack of knowledge than from emotional barriers such as fear and uncertainty. Avoiding trial-and-error learning hindered their development of independent skills.

Overall, despite device ownership, older women in the pre-DCTP period lacked the ability to use them independently, effectively, and securely—a convergence reflected in both learners’ accounts and policymakers’ assessments. Importantly, these findings raise a key question: why did older women feel the need to strengthen their digital capital? Their motivations were shaped not only by deficits in ownership and competence but also by expectations of how digital skills could enhance their everyday lives. These motivations are explored in the next theme.

### 3.2. Theme 2: Motivations for Enhancing Digital Capital

In this theme, digital capital is considered not merely as a set of technical resources, but as a motivating force shaping individuals’ desire to participate in digital society. In this respect, while Theme 1 focused on learners’ existing capacities, Theme 2 explores their motivations for further digital engagement. In this thematic analysis, the motivating factors for enhancing digital capital are grouped under four sub-themes: (2a) adapting to the digital society, (2b) maintaining family roles and social status, (2c) striving for individual independence and autonomy, and (2d) sustaining social ties and overcoming isolation. While the sub-themes are presented separately, participants’ experiences frequently intersect across these boundaries; therefore, the fluidity between sub-themes is highlighted.

#### 3.2.1. Sub-Theme 2a: Adapting to the Digital Society

For the majority of learners (n=10), the main motivation was not to fall behind in a world where digital tools increasingly shaped everyday life. Their age group (60–78) aligned with Prensky’s [25] concept of ‘digital immigrants,’ making adaptation feel more urgent in contrast to ‘digital natives’ in their families. Many participants stated that they did not want to appear ignorant or dependent in front of their children or grandchildren. Digital competence was not seen as a luxury reserved for the young, but rather as an everyday necessity—“a requirement of our time,” as several learners put it. For these participants, adapting to a digital society involved not only acquiring technical skills but also learning to navigate digital services securely. This need was clearly illustrated in L4’s words:

“Personal information such as our credit card or pension card can be accessed through various platforms. I find this environment very unsafe and I’m afraid of making mistakes. However, still, we have to use these systems. It has become a necessity to handle transactions on the phone instead of going to the bank.”

(L4)

For the majority of learners (n=10), digitalization was perceived as inevitable. Yet adaptation was accompanied by security concerns and fear of mistakes. Thus, while highlighting their motivation to keep up with the demands of the era, learners simultaneously expressed their desire to manage this process safely. In a similar vein, the policymakers framed digitalization as a “requirement of the era,” while the educator emphasized not only women’s access to tools but also the importance of using them securely and autonomously. Consequently, for both learners and planning actors, adapting to the digital society emerged not merely as a matter of technical competence but as a vital necessity in terms of social belonging, security, and visibility. Taken together, these perspectives reveal a shared understanding across learners, the educator, and the policymakers regarding adaptation to digitalization as a matter of security, autonomy, and social belonging.

#### 3.2.2. Sub-Theme 2b: Preserving Family Roles and Social Status

The preservation of social roles and status emerged as another key sub-theme in learners’ processes of acquiring digital skills. They felt the need to gain digital competences in order to maintain their roles within the family, to appear knowledgeable in front of their children and grandchildren, and to avoid losing social respect. L10’s account illustrates this clearly:

“I always supported their education; I kept their books, listened to what they explained, and stood behind them. Now they all have professions and are moving forward in their own lives. However, now it is me who has to catch up with them. They use the technology in their hands so effortlessly; I, too, strive to keep pace with them, to avoid falling behind in their world.”

(L10)

This account illustrates that women, due to their limited digital capital, perceived generational gaps within the family more acutely and believed this undermined their social standing. The majority of learners were mothers and many were also grandmothers; these familial roles heightened the pressure to acquire digital skills. In addition, some trainees reported that even seemingly minor digital shortcomings could lead to a loss of prestige in their social circles, highlighting how critical digital capital was perceived to be for maintaining both familial and social status. The policymakers and the educator reported similar observations. In their view, older women’s limited digital skills not only hindered their ability to manage everyday tasks independently, but also weakened their roles within the family. The educator emphasized that women’s dependence on their children and grandchildren for digital help undermined their authority within the household. Instead of being the transmitters of knowledge, women were pushed into the position of constantly requesting assistance. The educator explained this dynamic as follows:

“Participants often shared that they had to rely on their grandchildren for digital help—a situation they found disempowering. The grandchildren, viewing it as a burden, would sigh or take the device and complete the task themselves, leaving the women feeling dependent and excluded”

(E1)

This situation negatively affected older women’s respect and standing within the family. One policymaker stressed that digital skills were critical for preserving older women’s social status and maintaining active roles within the household:

“Digital competence is more than a technical skill; it is a tool for exercising basic rights. For an older individual, preserving one’s place and respect in social life is possible only through access to these rights. Digital capital is the precondition for sustaining one’s position as a full member of society.”

(PM1)

Such accounts highlight that older women regarded digital capital as essential for sustaining their societal roles, preserving social status, and ensuring intergenerational respect—far beyond its functional use. This view was consistently echoed across the narratives of learners, the educator, and the policymakers. Closely related to these concerns about status was the pursuit of individual independence, which emerged across participant groups as equally central to motivation.

#### 3.2.3. Sub-Theme 2c: The Pursuit of Individual Independence and Autonomy

The pursuit of individual independence emerged as one of the strongest motivations for all learners’ participation in the course. They described being able to carry out digital transactions without needing assistance from others as an essential part of their autonomy. L4’s statement clearly reflected the unsettling nature of such dependency:

“People in our age group, especially if they don’t have children, can become dependent on others in many matters. Who should I go to for help? The young employees at the bank knew all my passwords; I had to trust them. This really disturbed me because I couldn’t do anything on my own …”

(L4)

Similarly, the policymakers and the educator noted that older women lacking digital capital were unable to adapt to the digital world, which left them dependent on others and restricted to the timing and priorities of those from whom they sought help. The educator (E1) reported that many learners had to rely on family members even for basic tasks such as booking hospital appointments or handling banking transactions, but that sufficient support was not always available. One policymaker (PM2) explained that the inability to perform digital transactions undermined individuals’ independence:

“So many things are now done through digital platforms. When there is no one nearby to provide support, these people cannot participate in the world or in communication. Therefore, we decided to support them in this regard.”

(PM2)

These findings reveal that the lack of digital capital limited older women’s ability to carry out everyday digital tasks—including accessing services, communicating, and navigating online environments safely. As such digital skills are now essential for daily life, the absence of these competences reinforced a condition of dependence, leaving older women reliant on others to navigate the digital aspects of everyday life.

#### 3.2.4. Sub-Theme 2d: Sustaining Social Ties and Overcoming Isolation

The shift of social relationships increasingly onto online platforms made the acquisition of digital skills an important source of motivation for learners. They felt the need to be active in the digital world in order to maintain their social visibility and sustain their connections with close circles. L1 explained this motivation as follows:

“I joined this course to stay in touch with people I know. Nowadays everyone is in the digital environment, and I realized that I had to take part in the digital world in order not to lose connection with them.”

(L1)

Similarly, L12 expressed her desire to move beyond her limited use of social media and to become a more visible and interactive individual:

“I was able to share on Facebook, but I didn’t know how to post on Instagram. That is why I joined the course. I wanted to be a more social and more visible person.”

(L12)

The observations of the educator and the policymakers supported these accounts. In their view, the lack of digital skills led not only to individual isolation but also to the weakening of ties with social networks. PM2 described this situation as follows:

“Communication is a big part of life today. To stay connected, you need to use technology-everything is online now. However, older women really struggle with this, and that is why they end up feeling cut off and alone. Wanting to make life easier and keep in touch with loved ones was their main motivation.”

(PM2)

Across all narratives in this theme, digital capital emerged not merely as a set of functional skills, but as a resource intertwined with aspirations for social respectability, relational belonging, and independent action. As consistently emphasized by learners, the educator, and the policymakers, motivations extended beyond technical goals and reflected a deeper need for agency and recognition. This framing offers a conceptual bridge to the next theme, which explores the outcomes of digital engagement.

### 3.3. Theme 3: Gains–Toward the Effective Utilization of Digital Capital

This theme examines the impact of the DCTP on older women’s digital competences through a tripartite actor perspective encompassing learners, the educator, and the policymakers. As discussed in Theme 1, older women’s levels of digital ownership were limited prior to the programme, and this situation largely persisted after its completion. Following the withdrawal of the tablets provided during the training, smartphones remained the primary means through which older women accessed the digital world. Accordingly, the DCTP did not lead to a sustained increase in digital device ownership or diversification.

Despite these constraints, the programme made a substantial contribution to how older women used their existing devices. Data from all three actor groups indicate a notable increase in learners’ digital competences, reflecting more effective, conscious, and purpose-oriented use of predominantly smartphones. A comparison of digital competences before and after the DCTP, which combined practice-oriented training, repeated use of familiar devices, and task-focused modules addressing everyday digital needs, reveals clear improvements across all five competence domains.

Prior to the programme, information and digital competence skills were observed among nine of the 13 learners; this number increased to 11 following the training. Competence in communication and collaboration rose from 10 to 12 learners, while digital content production skills increased from five learners before the programme to 7 afterwards. The most pronounced gains were observed in security and problem solving. Whereas none of the learners demonstrated competence in digital security prior to the training, five learners did so after the programme. Similarly, problem-solving competence increased markedly, from one learner before the DCTP to seven learners following its completion. Overall, although digital ownership remained largely unchanged, the findings demonstrate that the DCTP resulted in measurable improvements in older women’s digital skills, particularly in domains where baseline competence levels were lowest. These skill gains provide the foundation for understanding how digital competences were subsequently translated into broader outcomes related to independence, visibility, and social participation, which are examined in the following sub-themes.

#### 3.3.1. Sub-Theme 3a: Personal Empowerment

This sub-theme illustrates the psychological impact of digital capital, illustrating how the competences acquired through the DCTP translated into greater self-confidence, autonomy, and a reduced fear of engaging with digital technologies. It focuses on trainees’ growing capacity to act more independently in their daily lives and to perceive themselves as capable digital actors. Although digital device ownership and diversity remained limited, the combined development of skills across the five domains of digital competence, together with more effective use of existing devices, led to notable psychological gains. Several learners reported being able to complete tasks they had previously been unable to manage without assistance—such as online banking or accessing e-government services—and described a greater ability to manage daily routines with reduced dependence on others. As this learners increasingly managed digital responsibilities on their own, they reported a growing sense of reduced dependence on family members and a strengthened feeling of autonomy. This capacity for independent action was closely accompanied by an internal shift–these learners described enhanced confidence in their own digital capabilities and a greater belief in their ability to navigate digital spaces without external assistance. This capacity for autonomous action was accompanied by an internal shift. Several learners reported increased confidence in their digital abilities and a stronger belief in their ability to navigate online environments without external help. This growing sense of self-efficacy contributed to the gradual reduction of hesitation, anxiety, and fear—emotions that had previously shaped their digital experiences. Over time, these feelings were replaced by a more robust sense of competence and self-assurance. As one learner reflected:

“I feel more knowledgeable and secure now. When a friend needs help with something, I can say, ‘Let me show you, I know how to do it.’”

(L13)

Observations from both the educator and the policymakers further supported the learners’ accounts, highlighting that the programme’s impact extended beyond technical learning to broader experiences of independence and empowerment. The educator noted that even when the learners did not fully master every digital task, gaining the confidence to explore and act on their own represented a significant outcome. Similarly, a policymaker (PM1) emphasized a learner’s statement—“I am no longer dependent on my children”—to illustrate how increased digital confidence translated into a broader sense of autonomy, enabling older women to manage everyday tasks independently and to rely less on others in both family and daily life contexts.

#### 3.3.2. Sub-Theme 3b: Social Ties

This sub-theme highlights the social dimension of digital capital by examining how the digital competences developed through the programme were translated into strengthened social ties, increased visibility, and recognition within family and community contexts.

For some learners (n=3), participation in the programme facilitated entirely new forms of social connection, while for others (n=11), it expanded and reinforced existing communication practices. These developments supported sustained contact with geographically distant family members, an outcome that the learners described as particularly meaningful given their roles as mothers and grandmothers. Beyond online interaction, the face-to-face structure of the programme played a crucial role in fostering social bonds and generating social capital in familiar senior centers, where women learned together, strengthened peer relationships, and formed new friendships. As one learner noted:

“I joined the course to learn digital skills, but I also made friends there. The environment was wonderful, and spending time together did me a lot of good.”

(L13)

Digital engagement also reshaped perceptions of social status and recognition. Some learners (n=4) reported that their increased digital engagement earned them greater respect from family members and peers. As one participant explained:

“My children especially appreciate it. My friends even nicknamed me the ‘technology professor.’”

(L10)

At the same time, small number of accounts reveal that digital engagement could also produce new dynamics of social comparison. Differences in learning pace and outcomes shaped how participants evaluated themselves in relation to others, sometimes generating feelings of prestige and, at other times, perceptions of inadequacy. As one learner expressed:

“Some of our friends really learned well… I, on the other hand, joined mostly for appearances, and it didn’t really benefit me.”

(L5)

Observations from the policymakers and the educator further supported these patterns. One policymaker illustrated this shift in social visibility and family dynamics as follows:

“When a woman takes the tablet home, the atmosphere in the household changes. We are also challenging family perceptions. Grandchildren no longer say, ‘My grandmother does not know anything’; instead, they say, ‘My grandmother has a tablet.’ This transforms the relationship between grandmother and grandchild; the nature of the relationship changes. The learners told us this themselves.”

(PM1)

The policymakers noted that many women participated not only to develop digital abilities but also as a way of coping with loneliness and social isolation. In a similar vein, the educator observed that the learners often extended the social dimension of the programme beyond the classroom by sharing what they had learned with peers and neighbours, thereby building new social connections. This diffusion of knowledge increased their visibility and, in some cases, positioned them as informal role models within their communities. Overall, the findings show that the digital competences developed through the programme were converted into strengthened social ties, increased recognition, and enhanced visibility in both family and community settings. These social outcomes illustrate how digital capital operates not only as an individual resource but also as a relational asset that reshapes older women’s positions within their social environments.

### 3.4. Theme 4: Deficiencies and Barriers

#### 3.4.1. Sub-Theme 4a: Content and Methodological Limitations

One of the most frequently mentioned areas for improvement concerned the content and delivery of the training. As reflected in the learners’ accounts and corroborated by the observations of the educator and the policymakers, these shortcomings manifested at different levels. The findings concentrated particularly on two dimensions: limitations in content and limitations in methodology. The most frequently emphasized limitation was that the course content was not sufficiently adapted to everyday needs. Several women (n=5) stated that the lessons were too theoretical and did not cover practical daily tasks, while others (n=4) emphasized that the curriculum lacked flexibility to address individual needs:

“I wanted to manage without depending on anyone, but the course ended before covering practical needs.”

(L12)

Both the policymakers and the educator agreed with the learners that the content should be directly linked to daily life, personalized, and needs-based. The educator noted that learners’ demands were taken into account—for example, pre-designed modules were updated in line with the questions and requests raised by trainees—though not all expectations could be met. A policymaker (PM2) summarized the rationale for this approach as follows:

“It is crucial to conduct a proper needs analysis. As local governments, sometimes we implement activities that are not really needed. This leads to a waste of both time and resources …When the analysis is done properly, everyone benefits.”

Another frequently mentioned shortcoming was the insufficient coverage of digital security. Several learners (n=4) reported that they expected more detailed guidance on online shopping, banking transactions, and data protection. Their accounts revealed that they did not feel confident in managing these practices on their own, leading them to approach online shopping and banking with great hesitation or to avoid such practices altogether. The educator’s narrative confirmed the learners’ concerns that individual security issues were insufficiently addressed, while also highlighting that a more holistic digital ethics perspective—including privacy and collective responsibility—was almost entirely absent.

Class size and structure were also cited as challenges. Although the limited class size of ten learners was viewed positively, differences in learning pace and participant profiles still posed difficulties. The policymakers had assumed a relatively homogeneous group with similar disadvantages, and designed the content and methodology accordingly. However, the educator’s experience revealed a heterogeneous group, differing not only in terms of digital capital but also in learning styles, pace, and pedagogical needs. The educator emphasized the difficulty of teaching such diverse learners simultaneously, while the learners similarly noted that age-related learning difficulties were not taken into account and that information was not delivered in ways sensitive to cognitive differences. They stressed that the program should be reconsidered not only by class size but also by group diversity.

All three actors highlighted the importance of an interactive learning environment for motivation. Face-to-face lessons were considered strong in terms of question-and-answer sessions and peer learning, and this environment was noted to enhance motivation. However, both the educator and some trainees (n=4) reported that this dynamism was lost in online sessions. Overall, participants described the program content as insufficiently needs-based, security-focused, and sensitive to individual differences, but emphasized that the interactive learning structure was one of its strongest aspects.

#### 3.4.2. Sub-Theme 4b: Limitations of Access and Inclusivity

The narratives revealed that barriers to the sustainability and inclusivity of the training were intertwined across individual, structural, and institutional levels. The policymakers most frequently emphasized the lack of devices, weak internet connections, and limited venue availability; PM1 regarded these not merely as technical but structural barriers, stressing that sponsorships and multi-stakeholder collaboration were essential for sustainability. The educator similarly noted that shortcomings in basic equipment and online delivery reduced both the effectiveness of the lessons and the level of interaction. Although the learners did not directly mention these problems, their demands for the reopening of the program and for training to be offered in closer venues indirectly reflected the difficulties created by such conditions. In particular, three learners stressed that attending courses in distant centers was challenging and underlined that local venues were critical for continuity.

A significant number of learners (n=5) reported that the digital skills they had acquired were forgotten over time or at risk of being forgotten; some said they had already forgotten what they learned, while others described developing personal strategies to retain knowledge. For this reason, they requested repeated sessions and written support materials (e.g., booklets):

“We are all old, we forget. It would be better if small booklets were prepared …”

(L1)

Although the policymakers and the educator did not directly address this issue, several trainees (n=6) stated that they had already forgotten what they learned or were at risk of forgetting. Nine learners emphasized that the program should not remain a one-off initiative, with some highlighting the risk of forgetting as a reason for re-enrollment. In addition, inclusivity emerged as another concern. Some learners (n=4) found it limiting that the program was designed exclusively for older women and argued that similar training should be extended to broader groups. The educator highlighted that domestic responsibilities—particularly caring for grandchildren—restricted women’s regular participation, noting that without flexible scheduling and alternative delivery models, inclusivity would remain constrained. The policymakers also recognized the need to expand training to wider audiences but underlined that such a goal could not be achieved without multi-stakeholder support mechanisms.

When the narratives of learners, the educator, and the policymakers are considered together, it becomes evident that limitations of access and inclusivity were not only technical or individual in nature but directly linked to structural and institutional conditions. While the learners called for more locally accessible and continuous programs, the educator pointed to barriers rooted in technology and gender roles, and the policymakers emphasized the necessity of multi-stakeholder support mechanisms for sustainability. The policymakers and the educator stated that, without stronger multi-stakeholder support mechanisms, the prestige and visibility gains identified in Theme 3b largely remained limited to learners’ close social environments. The narratives reflect that digital capital was shaped not only by individual efforts but also by the opportunities and support mechanisms provided by the wider system. In sum, the DCTP was described as enhancing learners’ digital skills, self-efficacy, and social visibility, yet participants also reported persistent limitations related to content, security, retention, and structural support.

## 4. Discussion

This study examined older women’s experiences of digital competence training through the lens of digital capital theory, highlighting transformations at individual, social, and structural levels. Findings show that digital capital extends beyond technical skills, encompassing self-efficacy, social ties, visibility, and structural support. In this sense, the study both resonates with existing literature and offers original insights within the Turkish context.

Most learners reported increased confidence in using digital tools and greater independence in daily life. This supports digital capital theory’s claim that individual resources—knowledge, skills, and self-efficacy—shape social participation [20]; while previous studies have similarly linked digital competence to enhanced autonomy and self-esteem [26,27,28,29,30,31], this study further highlights the emotional and psychological transition participants undergo. The learners’ experiences were initially shaped by fear, anxiety, and insecurity, including concerns about making mistakes, causing harm, or being exposed to online risks. These emotions were frequently intertwined with feelings of shame, perceived inadequacy, and dependence on others. As digital capital accumulated, the learners increasingly reframed their engagement with digital technologies as manageable, meaningful, and socially embedded. This shift was accompanied by growing confidence, enhanced self-efficacy, and a transformed self-perception as capable digital actors. In this sense, our study highlights that digital capital operates not only as a set of technical competences but also as a psychosocial resource, facilitating emotional security, confidence, and a sense of capability in later life. Building on this psychosocial dimension, our study extends this body of work through a gendered lens. In the Turkish context, increased digital competences emerged as crucial resources of empowerment for older women, enabling them to renegotiate family roles, reduce dependency, and gain recognition as capable and autonomous actors. Digital skills thus function not only as individual learning outcomes, but as forms of digital capital that intersect with and potentially challenge gender-based inequalities.

Trainees increasingly used digital tools to communicate with family members and friends, thereby strengthening their social ties. Previous studies have likewise shown that digital communication can reduce loneliness and foster social participation among older adults [32,33]. Our findings extend this literature by demonstrating that social bonds were reinforced not only through online interaction, but also through the face-to-face learning process itself. Friendships formed within the training, along with reciprocal learning and experience sharing, generated enduring social connections and a sense of belonging among trainees. Although such interactional dynamics are acknowledged in international research, they have often been treated as secondary outcomes of digital training programmes [31,34]. More recent empirical studies, however, increasingly emphasize that digital competence should be understood beyond simple skill acquisition, highlighting the central role of supportive and continuous learning environments in fostering self-efficacy, motivation, and sustained engagement with technology [35,36,37]. Contributing to this emerging line of research, the present study demonstrates how these dynamics unfold in the Turkish context. Building on earlier national research that pointed to the importance of active participation, peer interaction, and mutual learning in competence-based digital education [38], this study shows more clearly how collective, active, and ongoing learning experiences generate psychosocial gains and foster community and social belonging among older women. By comparing the experiences of multiple actors—learners, educators, and policymakers—the analysis provides a more comprehensive and grounded account of digital competence training as a socially embedded and participatory process rather than merely a skills-based intervention.

A salient aspect of learners’ experiences was the transformation of digital competences into social prestige within family and community contexts. The learners described a shift from initial fear and embarrassment to growing confidence and autonomy in managing everyday digital practices, such as independently sharing locations or handling banking transactions. This reduced reliance on family members and enhanced recognition within close social relations. Earlier studies showed that older adults can renegotiate social roles through technology, yet gender is often treated as secondary or linked mainly to motivation and well-being [39,40,41]. Our findings extend this by showing that older women enhance visibility and renegotiate family roles through digital skills. However, digital competences also create new, subtle hierarchies, as some women felt inadequate when comparing their progress with peers. This dual process underscores the symbolic nature of digital capital—fostering recognition while generating differentiation [20,23,42].

While the program generated notable gains at individual and social levels, the findings also reveal that persistent structural constraints limited the extent to which these gains could be sustained and expanded. Limited space, inadequate equipment, and weak institutional support indicate that digital inclusion cannot rely on individual effort alone. Rather than reflecting temporary logistical shortcomings, these constraints point to the institutional and infrastructural dimensions of broader digital inequality, where unequal access to resources and support structures systematically limits participation. This interpretation resonates with recent empirical research demonstrating that well-being, social participation, and empowerment in later life are strongly shaped by contextual and environmental conditions rather than individual motivation or capability alone. For example, studies focusing on neighbourhood environments show that mobility-supportive and socially enabling local contexts play a critical role in sustaining social relationships and well-being among older adults [43]. Although such research does not address digital inclusion directly, it underscores a broader insight that empowerment in later life is contingent upon supportive structural and local conditions. In line with this perspective, our findings show that even relatively advantaged older women encountered difficulties in translating newly acquired digital competences into sustained digital engagement when institutional support remained limited. These barriers reflect intersectional forms of digital inequality, as training content and delivery often failed to fully align with participants’ age-related cognitive and physical changes, diverse educational backgrounds, and spatial conditions, rendering digital education formally available but substantively inaccessible for many older women. Such design mismatches are closely tied to institutional and policy-level arrangements governing resource allocation, programme ownership, and infrastructural support. Prior studies likewise stress that sustainable digital education depends on institutional ownership, stable funding, and technical infrastructure [44,45], underscoring that digital inequality extends beyond access to encompass conditions of use and long-term support. In Türkiye, such deficits remain major barriers limiting older women’s participation, illustrating how digital inequalities are reproduced in context-specific ways through structural and institutional arrangements. Taken together, these findings confirm theoretical perspectives emphasizing that digital capital is shaped not only by individual skills but also by the availability of structural support [20,42].

A key contribution of this study lies in its multi-actor approach to analyzing digital education processes. By incorporating the perspectives of learners alongside those of the educator and the policymakers, the analysis moves beyond a single experiential viewpoint and enables comparison between intended programme goals and lived learning experiences. Rather than treating these perspectives as confirmatory, the multi-actor design made it possible to identify tensions, gaps, and mismatches between how digital competence training was planned, how it was implemented, and how it was actually experienced by older women. While existing research on older women’s digital experiences has largely focused on individual-level accounts [39,40,41], this study demonstrates the analytical value of examining digital education as a process whose effects cannot be assessed solely through programme design or good intentions. Through this comparative perspective, the study offers a more grounded understanding of how a digital competence training programme aimed at building digital capital shapes, supports, or limits older women’s opportunities to acquire and use digital resources in their everyday lives.

It is also important to consider how the reliance on self-reported narratives shaped the interpretation of digital change in this study. The learners’ accounts often reflected how they perceived their own digital development, which in some cases did not align with objective indicators of digital competence. For example, a participant may describe themselves as “more independent” after gaining confidence in messaging friends on WhatsApp, even if their overall digital skill-set remained limited. Conversely, another participant may demonstrate high functional proficiency yet report no meaningful learning due to perceived misalignment with their personal goals. These discrepancies highlight that digital competence, especially in later life, is not solely about task completion but also deeply rooted in individuals’ subjective interpretations of what counts as meaningful, useful, or empowering digital engagement. Thus, while self-report data presents inherent limitations, it also provides a valuable window into the personal meanings attached to digital participation.

### 4.1. Policy Implications

The findings indicate that digital inclusion policies should move beyond a narrow focus on technical skills and instead adopt a holistic, intersectional, and community-based approach. The fact that, in this study, even relatively advantaged older women—characterized by urban residence, higher educational attainment, and stable income sources—experienced digital barriers underscores the urgency of designing policies that explicitly target groups facing compounded disadvantages. Community-based digital education initiatives should therefore be tailored to the heterogeneity of older populations by simultaneously accounting for age, gender, socio-economic status, ethnicity, and geographical location. In practice, this requires locally embedded and flexible program designs, such as neighbourhood-based learning spaces, mobile training units, or partnerships with community centers, particularly in rural or socio-economically deprived areas. For older women with low educational attainment or limited literacy skills, digital education should be integrated with basic literacy and everyday life competencies, ensuring relevance to daily needs. Such contextualized approaches can help mitigate multi-layered inequalities that cannot be addressed through standardized training models. Moreover, digital competence should be framed as a social right rather than an individual responsibility associated with neoliberal notions of “active aging”. From a policy perspective, this entails providing not only training but also structural support, including access to devices, affordable internet connectivity, and ongoing technical assistance through public or local government programmes. The collaborative model observed in this study—where private sector actors provided equipment, universities contributed theoretical frameworks, and local authorities offered implementation spaces—illustrates the potential of institutionalized public-private-academic partnerships. Embedding such collaborations within formal policy frameworks can enhance sustainability and prevent reliance on short-term project funding. Finally, community-based initiatives should prioritize social support mechanisms and peer learning structures to strengthen social capital and ensure the continuity of learning outcomes. Establishing neighbourhood-level digital hubs or peer mentoring systems can transform digital education into a collective process, reducing isolation and reinforcing mutual support among older women.

By foregrounding intersectionality, local embeddedness, and collective learning, digital inclusion policies can more effectively address the complex and layered disadvantages faced by older women. From an implementation perspective, community-based digital education programmes should be designed as recurring and adaptive training cycles rather than one-off interventions, allowing older women to revisit content as technologies and personal needs evolve. While face-to-face instruction remains central for trust-building and engagement, programmes may be further strengthened through blended learning formats that combine in-person sessions with simple, online-supported materials. Providing recorded and revisitable content can help prevent learning loss and enable participants to practice skills at their own pace. These programmes require dedicated staffing structures, combining technical trainers with facilitators experienced in working with older women and vulnerable groups. Peer learning mechanisms, particularly those involving participants with similar life experiences or prior training exposure, can enhance motivation by fostering a sense of relatability and collective efficacy. Where appropriate, intergenerational learning formats—such as limited collaboration with younger volunteers or students—may complement peer-based models by offering additional technical support without undermining participants’ sense of autonomy. In addition, digital safety and privacy modules should be embedded as a core component of all training activities, addressing common concerns related to online fraud, data protection, and fear of making mistakes. Institutionalizing these elements within public policy frameworks can strengthen sustainability and support long-term digital autonomy.

### 4.2. Limitations

This study has several limitations. First, the sample of older women was relatively small and confined to a specific geographical context, limiting the generalizability of the findings. In addition, only older women who attended older adult centers were included, as attendance at these centers was a requirement for participation in the DCTP. Consequently, the sample may represent a relatively active and socially engaged subgroup of older women, potentially representing only a small segment of the wider older female population. Second, analysis relied solely on participants’ narratives, creating potential subjectivity in interpretation. Third, the effects of the program could not be assessed longitudinally; as conclusions regarding learning retention and sustainability are primarily based on participants’ perceptions. While analytic consistency was strengthened through collaborative coding and consensus-building among researchers, formal statistical intercoder reliability measures were not calculated. Finally, although the qualitative design enabled in-depth exploration of participants’ experiences, the absence of quantitative measures limited the comparative strength of the findings.

### 4.3. Future Research

Future research should examine older women’s digital experiences cross different socio-economic and cultural contexts to better capture variations in digital opportunities and constraints. In particular, studies that account for socio-economic differences such as educational attainment, income levels, urban–rural settings, and traditional–modern social contexts would help clarify how structural and cultural conditions shape digital inclusion processes. An intersectional analytical perspective is therefore essential for understanding how these contextual dimensions interact rather than operate independently.

In terms of research design, future studies could benefit from comparative approaches that examine both programme participants and non-participants to better isolate programme effects. Independently of this, longitudinal research designs would strengthen evaluations by enabling assessments of learning retention, sustainability, and longer-term digital engagement beyond the immediate training period.

Methodologically, future research should adopt explicit mixed-methods designs that combine qualitative insights into subjective experiences—such as confidence, motivation, and perceived empowerment—with quantitative measures of observable digital competencies. Within such designs, task-based or standardized pre–post assessments may be used to provide concrete evidence of changes in participants’ ability to perform specific digital practices (for example, using social media platforms or accessing online services), allowing programme outcomes to be evaluated beyond self-reported perceptions and strengthening the empirical basis of digital inclusion research.

## 5. Conclusions

This study examined older women’s participation in digital competence training through the lens of digital capital theory, demonstrating that the gains extended beyond technical skills to include transformations in social ties, visibility, and community participation. At the same time, limitations related to sustainability, structural support, and inclusiveness emerged as key constraints on the continuity of digital engagement. These findings highlight that digital competence training alone is insufficient to ensure long-term digital inclusion for older women when broader structural conditions remain unaddressed.

Building on these findings, the study indicates that digital inclusion policies need to move beyond a narrow focus on technical skills and address broader social and structural inequalities. In particular, strengthening institutional collaboration, promoting sustainable training models, and integrating a gender-sensitive perspective into digital inclusion policies emerge as key considerations for enhancing older women’s digital participation.

Beyond its policy implications, this study makes a distinct contribution to digital capital and gender studies by demonstrating that digital capital operates not only as an instrumental resource for accessing services and information, but also as a relational and symbolic resource shaping older women’s social status, visibility, and sense of belonging. In this respect, the findings extend Ragnedda and Ruiu’s [20] conceptualization of digital capital by showing how its utilization intersects with age, gender, and family roles in later life. This dual function of digital capital underscores that digital competence should be analyzed not merely as a technical skill set, but as a form of capital embedded in social structures and capable of reproducing or transforming inequalities.

## Data Availability

The data presented in this study are available on request from the corresponding author due to privacy, ethical, and confidentiality considerations associated with human participant data.
